# Surgical treatment of displaced isolated lateral malleolar fractures: incidence of adverse events requiring revision: a retrospective cohort study

**DOI:** 10.1186/s13018-022-03135-z

**Published:** 2022-05-03

**Authors:** Jonas Ordell Frederiksen, Catarina Malmberg, Dennis Karimi, Peter Toft Tengberg, Anders Troelsen, Mads Terndrup

**Affiliations:** Department of Orthopedic Surgery, Copenhagen University Hospital Hvidovre, Kettegårdsalle 30, 2650 Hvidovre, Denmark

**Keywords:** Ankle fractures, Stability-based classification, Talar shift, Isolated lateral malleolar fractures, Adverse events

## Abstract

**Background:**

Recent systematic reviews support that non-operative management should be the standard treatment for all stable isolated lateral malleolar fractures (ILMFs), regardless of fibular fracture displacement. Surgical fixation of ILMFs carries a risk of adverse events (AEs), and many patients will later require implant removal. We wanted to estimate the incidence of AEs requiring revision after surgical fixation of “potentially stable” displaced ILMFs before non-operative treatment became standard care in our department.

**Materials and methods:**

To identify patients with “potentially stable” ILMFs who had been treated surgically in a historical cohort, we retrospectively applied the stability-based classification system, introduced by Michelson et al., to a cohort of 1006 patients with ankle fractures treated surgically from 2011 to 2016. The primary outcome of this retrospective cohort study was the incidence of AEs that had functionally significant adverse effects on outcome and required revision in the first 12 months after surgery. AEs were graded and categorized using the Orthopedic Surgical Adverse Events Severity (OrthoSAVES) System.

**Results:**

The study population comprised 108 patients with “potentially stable” displaced ILMFs; 4 patients (3.7% [95% CI (0.1–7.3%]) experienced AEs requiring revision in the first twelve months after surgery. There were 5 additional patients (4.6%) with functionally significant AEs where revision surgery was not indicated within the first twelve months after surgical fixation. A further 5 patients (4.6%) had AEs managed in the outpatient clinic (grade II); 36 patients (33.3%) required secondary implant removal due to implant-related discomfort.

**Conclusions:**

Surgical fixation of ILMFs carries a risk of severe AEs, and many patients will subsequently need implant-removal procedures. Further prospective studies are required to ascertain whether non-operative treatment can lower the risk of AEs and the need for additional surgical procedures.

**Supplementary Information:**

The online version contains supplementary material available at 10.1186/s13018-022-03135-z.

## Background

Diagnosing clinically relevant instability is key when treating isolated lateral malleolar fractures[1–3]. The stability-based classification system by Michelson et al. describes an algorithm to differentiate unstable ankle fractures, which should be treated surgically, from stable fractures, which should be treated non-operatively [4]. According to the stability-based classification, an ankle fracture should be considered stable if it does not require reduction, is unimalleolar, and shows sufficient ligamentous integrity to secure anatomical alignment of the talus under the tibia [4]. In this study, we retrospectively applied the stability-based classification system to a cohort of 1006 patients with ankle fractures treated surgically to identify patients with “potentially stable” displaced isolated lateral malleolar fractures (ILMF) according to these stability criteria [4]. Our hypothesis was that a substantial proportion of patients with “potentially stable” fractures could have been managed non-operatively if a preoperative stress-test had been performed to determine stability.

ILMFs are the most common type of ankle fracture [5–7]. A growing body of the literature supports treating stable ILMFs non-operatively [2, 8–11]. Surgical fixation of ILMFs carries a risk of severe AEs and many patients will require subsequent implant removal procedures [6, 12]. In clinical practice, many orthopedic surgeons continue to use the degree of fibular fracture displacement as an indication for surgery [12, 13]. A survey study showed that 91% of the responding surgeons stated fibular displacement as critical when deciding between surgical and non-operative ILMF treatment [12]. Fibular fracture displacement alone does not seem to be a predictor of pathological ankle joint kinematics after injury [14]. The medial deltoid ligament complex is believed to be the most important stabilizing structure in ILMFs [3, 15–17]. Deltoid ligament incompetence can result in lateral displacement of the talus, defined as talar shift (TS), leading to altered ankle joint kinematics [15]. Patients with displaced ILMFs without apparent TS in diagnostic non-weightbearing, non-stressed radiographs could thus be described as “potentially stable". Elucidating instability requires radiographic examination under simulated physiological stress [18]. Manual manipulation during fluoroscopy or other stress-tests, such as the gravity stress view or weightbearing radiographs, have been proposed [18]. Not all institutions routinely perform diagnostic stress-tests and in our setting, displaced ILMFs were often treated with surgical fixation without diagnostic stress-testing.

The purpose of this study was to assess the risk of severe AEs after surgical fixation of potentially stable displaced ILMFs. The objective was to estimate the incidence of AEs with functionally significant adverse effects on outcome that required revision in the first 12 months after surgery using standardized AE reporting. We hypothesized that the incidence of AEs requiring revision within the first 12 months would be below 10%.

## Materials and Methods

Permission to assess patient information was granted by the Danish Health Authority (j.nr 3-3013-765/1). Approval from the data protection agency was obtained prior to the conduction of this single center retrospective cohort study (j.nr.: 2012-58-0004). The reporting conforms with the “Strengthening the Reporting of Observational Studies in Epidemiology (STROBE)” checklist [19].

### The stability-based classification system

The stability-based classification system defines unstable ankle fractures as “(1) any ankle fracture dislocation, (2) any bimalleolar or trimalleolar ankle fracture, (3) any lateral malleolar fracture with a significant TS (usually more than 1 to 2 mm (mm) increase in medial clear space measured relative to the superior clear space)” [4].

### Displaced isolated lateral malleolar fracture definition

**Fig. 1:**
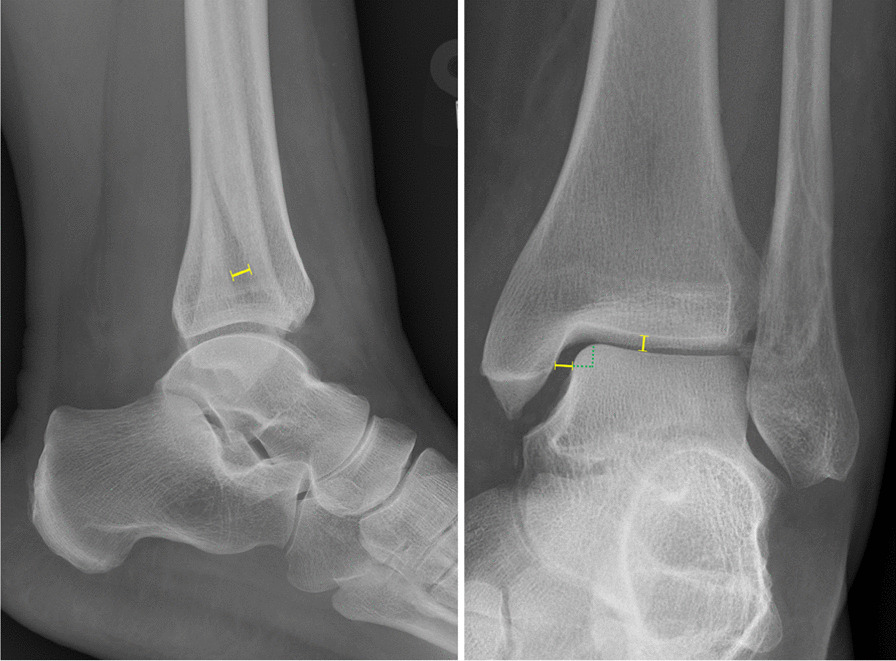
Radiograph with measurements. 1. Maximal fibular fracture diastasis, measured perpendicular to the fracture line, in any initial non-weightbearing, non-stress radiographic view (anterior/posterior, lateral or mortise). 2. Superior clear space (SCS), measured from the superior border of the talar dome at the highest point to the inferior border of the tibial plafond [21]. 3. Medial perpendicular clear space (MpCS), measured as the distance between the medial border of the talus and the lateral border of the medial malleolus, on a line parallel and 4 mm below the talar dome [22]

In this study, a displaced fracture was defined as a fibular fracture with a diastasis of more than 2 mm, measured in any initial non-weightbearing, non-stress radiographic view (anterior/posterior, lateral or mortise). “Potentially stable” ILMFs were ankle fractures that did not require reduction, did not involve the posterior or the medial malleolus, and were without apparent TS [4]. Apparent TS was defined as a 2-mm increase of medial clear space measured relative to the superior clear space in the mortise view of initial non-weightbearing, non-stress radiographs [12]. Clear space measurements were performed by two authors independently, using a described technique presented in Fig. 1 [20–22]. Measurements were performed with 0.1 mm accuracy using the DICOM digital ruler in Enterprise Viewer (Impax Client; Agfa-Gevaert N.V., Mortsel, Belgium).

### Standardized adverse event reporting

The Orthopedic Surgical Adverse Events Severity (OrthoSAVES) System provides a list of AEs that serves as a prompt for users and aims to standardize the AE terminology as well as the grading of AEs in orthopedic patients [23–25]. The different OrthoSAVES grades are defined in Table 1. Adverse events with functionally significant adverse effects on outcome, either temporary (less than 6 months grade III) or prolonged (more than 6 months grade IV, V and VI) were of interest in this study. The OrthoSAVES categories are presented in the Additional file 1: Appendix [26].

**Table 1:** Orthopaedic surgical adverse events severity [OrthoSAVES] system

Orthopaedic surgical adverse events severity system [25]	
Grade	Definition
I	Adverse event does not require treatment and has no adverse effect
II	Adverse event requires simple or minor invasive treatment (e.g., antibiotics, Foley catheter, nasogastric [NG]) tube) and has no long-term effect on patient outcome
III	Adverse event requires invasive (e.g., surgery) or complex treatment (e.g., monitored bed) and is most likely to have a temporary (less than 6 months) adverse effect on outcome
IV	Adverse event requires invasive (e.g., surgery) or complex treatment (e.g., monitored bed) and is most likely to have a prolonged (more than 6 months) adverse effect on outcome^1^
V	Sentinel or significant life or limb threatening event^2^
VI	Adverse event resulting in death

1. Any adverse event with functionally significant (i.e., patient-reported or objective) and most likely prolonged (> 6 months) adverse effect on outcome should be graded as severity grade 4, regardless of treatment complexity (or scenario where there is no possibility of treatment). 2. A sentinel event is an unexpected serious life or limb-threatening event and/or an event that necessitates institutional investigation and review to determine the root cause. For example, the wrong surgical site should automatically be graded at severity 5

### Primary outcome

Incidence of AEs with adverse effect on outcome (grade III or higher), requiring revision within the first 12 months after surgical fixation, reported as a binary variable.

### Exploratory outcome

- Different types of AE (grade III or higher) requiring revision within the first 12 months, reported as categorical variables defined in the OrthoSAVES system.
- All AEs in the cohort, categorized and graded using the OrthoSAVES system.
- Incidence of implant-removal procedures due to implant-related discomfort, performed more than 6 months after initial surgery, reported as a binary variable. Routine isolated syndesmotic screw-removal procedures were not categorized as an implant-removal procedure.

### Variables and data management

**Fig. 2:**
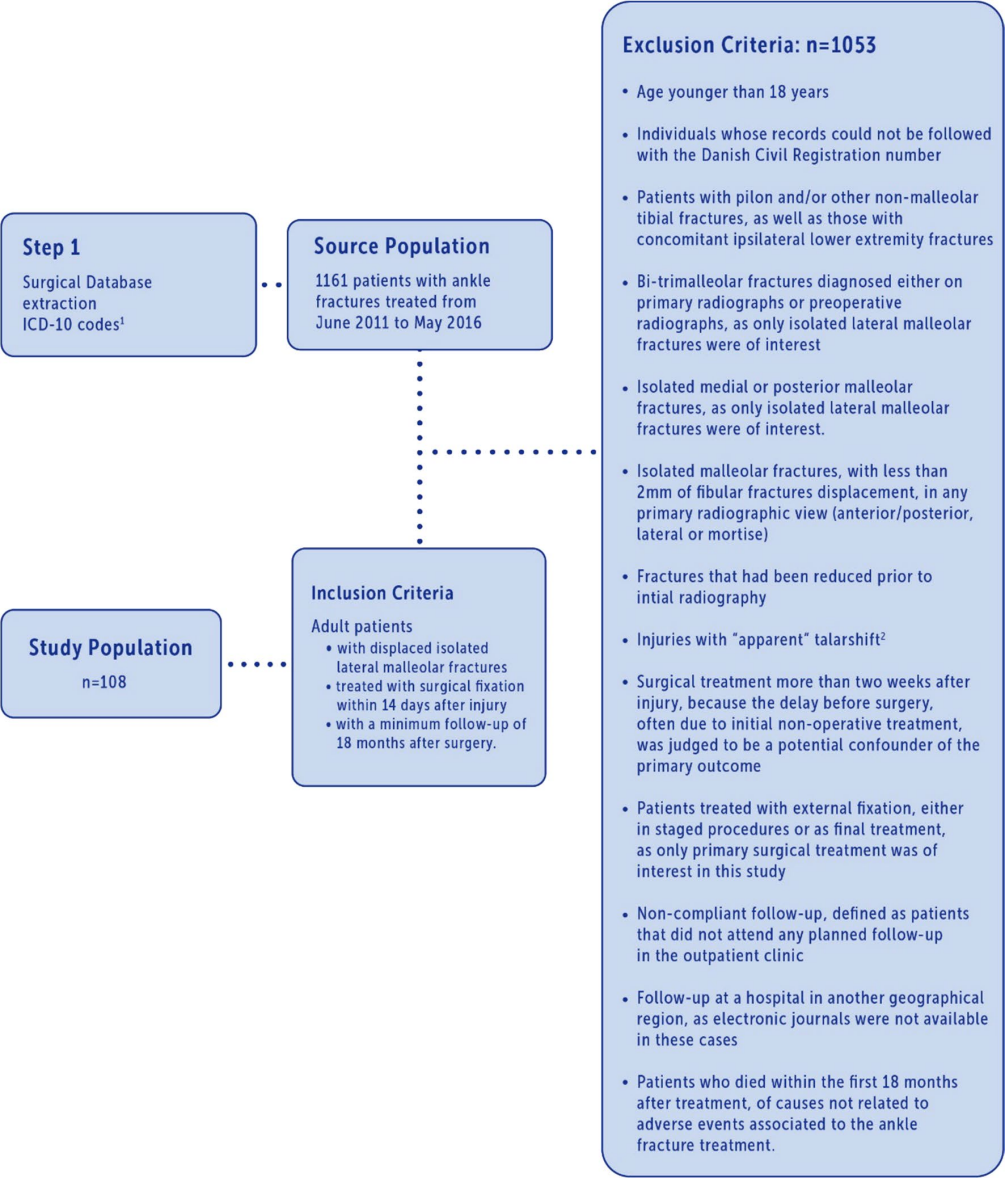
Study population. 1. 10th revision of the International Statistical Classification of Diseases and Related Health Problems: S82.3 Fracture of lower end of tibia; S82.4 Fracture of fibula alone; S82.5 Fracture of medial malleolus; S82.6 Fracture of lateral malleolus; S82.7 Multiple fractures of lower leg; S82.8 Fractures of other parts of lower leg; S82.9 Fracture of lower leg, part unspecified. 2. Apparent TS was defined as a 2 mm increase in perpendicular medial clear space measured relative to the superior clear space in the mortise view of initial non-weightbearing, non-stress radiographs [12]

The target population was adult patients (age ≥ 18 years) with displaced ILMFs. Patients were retrospectively sampled through review of electronic records of surgically treated patients from June 2011 to May 2016 at a single center, an academic level III trauma center serving 540,000 people. Since June 2016, non-operative treatment has been the standard care for these injuries at our institution. Records and radiographs were not available for patients treated before 2011. The source population was identified by querying the surgical database from June 2011 to May 2016. The diagnostic codes used to identify the source population and the inclusion/exclusion criteria in this study are presented in Fig. 2. Radiographs and surgical records were retrospectively reviewed by two authors simultaneously. Data were entered and managed in SPSS (Statistical Package for the Social Sciences, IBM Corp. IBM SPSS Statistics for Windows, Version 25.0. Armonk. NY. IBM Corp. 2017). Definitions of baseline variables are described in Table 2. The senior author re-reviewed all included patients. Registered AEs were categorized and graded by two authors independently, using the OrthoSAVES system. Discrepancies were discussed and co-authors were consulted if consensus could not be reached. Missing values were checked and collected if possible. Extreme values were proofed and corrected if applicable. The source population, and therefore the sample size, was restricted, as described above. All eligible patients were included.

**Table 2:** Baseline demographics and fracture classification

**Baseline characteristics**	
Sex (% male)	54 (50%)
Age in median years (range)	45.5 [18–82]
**ASA score**^**1**^** (no. of patients (% of study population)**	
1. Normal healthy individual	61 (56.5%)
2. Mild systemic disease not limiting activity	42 (38.9%)
3. Severe systemic disease limiting activity but not incapacitating	5 (4.6%)
Active Smoker (no. of patients (% of study population)	38 (35.2%)
Alcohol abuse^2^ (no. of patients (% of study population)	7 (6.5%)
Body mass index^3^ (mean SD [Range])	26.5 (± 4.3 [17.6–40.3])
Diabetes Mellitus (Type I or II) (no. of patients (% of study population)	5 (4.6%), all Type II
**AO classification**^**4**^** (no. of patients (% of study population)**	
AO44B1	96 (88.8%)
AO44C1	10 (9.3%)
AO44C2	2 (1.9%)
Median fibular fracture displacement^5^ (millimeters) (IQR, Range)	3,4 (1.8 [2. 1–8.9])
No. of patients with 2–4 mm displacement	76 (70.4%)
No. of patients with more than 4 mm displacement	32 (29.6%)

1. American Society of Anesthesiologists Classification (ASA Class) system for categorization of a patient’s physiological status [40]. 2. Alcohol abuse is defined as consuming more than 14 units per week for men, 7 for women [41]. 3. Body mass index defined as the body mass divided by the square of the body height, kg/m2 [42]. 4. Arbeitsgemeinshaft für Osteosyntesefragen (AO-) classification [28]. 5. Fibular fracture diastasis, as defined in Fig. 2

### Statistical method description

The primary outcome was recorded as a binary variable and is presented descriptively as a proportion. The confidence interval was estimated using the Wald method [27]. Exploratory outcomes were recorded as categorical data and presented descriptively with frequencies and proportions. Data were analyzed using SPSS (IBM Corp. Released 2017. IBM SPSS Statistics for Windows, Version 25.0. Armonk, NY: IBM Corp).

## Results

We reviewed 1161 patients for eligibility. Of 1006 adult patients undergoing surgical treatment of ankle fractures, 108 were classified as having potentially stable displaced ILMFs and were included in the study population, shown in Fig. 1. Baseline demographics and fracture classifications are presented in Table 2. The different surgical fixation techniques and postoperative plans are shown in Table 3. Of the patients, 96 (88.9%) had trans-syndesmotic type fractures and 12 (11.1%) suprasyndesmotic type fractures, classified as AO44B1 and AO44C1/AO44C2, respectively, using the Arbeitsgemeinshaft für Osteosyntesefragen (AO-) classification [28]. A semitubular plate was the implant of choice for fibular fracture fixation in 91.7% of cases. Locking compression plates, locking anatomical plates and intramedullary fibular nails were used in the remaining cases. Transsyndesmotic screw fixation was deemed indicated in 11 cases (10.2%), of which 7 had supra-syndesmotic fractures. Weightbearing as tolerated was permitted in 64 patients (59.3%) immediately after surgery.

**Table 3:** Internal surgical fixation technique, utilized implants and postoperative management

Surgical fixation technique	No. of patients (% of study population)
Lateral fixation only	95 (88%)
Lateral fixation + Syndesmotic fixation	10 (9.3%)
Syndesmotic fixation only	1 (0.9%)
Fibular Rod Fixation only	2 (1.9%)
Syndesmotic fixation with 1 or 2 trans-syndesmotic screws	11 (10.2%)
Intermedullary fibular nail with trans-syndesmotic screws	2 (1.9%)
**Postoperative bandage type**	
Orthosis (Walker boot)	29 (26.9%)
Circular Cast	67 (62%)
Change from circular cast to orthosis	12 (11.1%)
Immediate full weightbearing	64 (59.3%)
No weightbearing	36 (33.3%)
Partial weightbearing	8 (7.4%)
Patients restricted 2–4 weeks before full weightbearing	18 (16.7%)
Patients restricted 4–6 weeks before full weightbearing	26 (24.1%)
Patients restricted more than 6 weeks before full weightbearing	4 (3.7%)
Duration of immobilization in a bandage (median in days [Range])	43 [29–95]
Patients immobilized in a bandage up to 50 days	94 (88.7%)
Patients immobilized in a bandage more than 50 days	12 (11.3%)

Of the 108 patients, 4 (3.7% [95% CI (0.1%–7.3%]) experienced AEs grade III or higher requiring revision in the first 12 months after surgical fixation. Of these 4 patients, 2 had deep wound infections with positive biopsies, and 2 had implant-related AEs: aseptic loosening of a semitubular plate and malpositioned trans-syndesmotic screw fixation. All other AEs observed in the cohort are presented in Table 4. There were 5 patients (4.6%) with AEs (grade III or higher) not requiring revision within the first 12 months after surgery. Another 5 patients (4.6%) had superficial wound infections or wound dehiscence, managed with oral antibiotics and/or debridement in the outpatient clinic.

**Table 4:** All adverse events observed in the study population

All adverse events^1^ observed in the cohort	*n* (% cohort)	Grade II	Grade III	Grade IV
**Perioperative implant/fixation related**				
Malpositioning requiring revision	2 (1.9%)	0	0	2
Aseptic loosening	1 (0.9%)	0	0	1
**Infection**				
Superficial wound	3 (2.8%)	3	0	0
Deep wound	3 (2.8%)	0	0	3
**Postoperative pain**				
New onset (e.g., neuro-pathic pain,reflex dystrophy/or pain disorder)	2 (1.9%)	0	0	2
**Thromboembolic event**				
Deep vein thrombosis	1 (0.9%)	0	1	0
**Other**				
Wound dehiscence	2 (1.9%)	2	0	0
Total	14 (13%)	5	1	8

1. Adverse events are graded and categorized with The Orthopedic Surgical Adverse Events Severity System [26]

Secondary implant-removal was required in 36 patients (33.3%) a minimum of 6 months after surgery, due to implant-related discomfort. Implants were removed at a median of 15 months after primary surgery (range [6–61 months]).

## Discussion

The AE reporting terminology in orthopedic clinical studies is highly variable and inconsistent [25, 29]. AEs reported in the different clinical studies of ankle fractures cannot be analyzed and compared adequately [25]. Most studies in the literature examine the incidence of AEs after surgical treatment of supination-external rotation type fractures. However, these also include bi- or trimalleolar fractures which makes it difficult to assess the relationship between specific subcategories of ankle injuries and the rate of AEs [30]. Only limited data are available on the specific incidence of AEs after surgical treatment of ILMFs. Two randomized controlled trials have been published comparing surgical and non-operative treatment of ILMFs. The terminology used in these studies does not allow for direct comparison of incidence of AEs in our study. Sanders et al. [31] randomized 81 patients to either surgical or non-operative treatment. In the surgical group (*n* = 41), one deep wound infection (2.4%) required revision; 4 patients (9.8%) had superficial infections and 4 (9.8%) required implant-removal procedures. Mittal et al. randomized 160 patients to surgical or non-operative treatment. The analysis showed that in the surgical group (*n* = 72), 5 patients (7%) required further unplanned surgery, 2 (3%) had major infection, 11 had (15%) minor infection, 5 (7%) had neurological injury, and 5 patients had (7%) deep vein thrombosis. In a retrospective study by Richardson et al. 10.7% of patients treated with ORIF following a lateral malleolar fracture developed surgical site infection within one year after surgery [30].

The incidence of AEs found in the present study seems high. We hypothesize that a substantial proportion of the patients in the study population could have been managed non-operatively if evaluated with a diagnostic stress-test to ascertain optimal treatment prior to surgery. There is a large body of evidence that ILMFs should be treated non-operatively if the deltoid ligament is competent and prevents TS [2, 6, 8–11, 31–37]. Diagnosing clinically relevant instability is key when determining optimal treatment for these patients, as 1 mm of TS can significantly alter tibiotalar contact pressures and TS has been shown to be a predictor of poor outcome [12, 17]. Talar shift can be estimated using different radiographic tibiotalar clear space measurements [20]. Surgical treatment seems to be associated with higher rates of AEs compared with non-operative treatment [6, 31]. Many surgically treated patients require implant-removal due to discomfort [12]. Short- and long-term functional results do not seem to differ significantly after surgical or non-operative treatment of ILMFs [6, 31, 32, 38]. Surgical treatment is associated with higher costs compared with non-operative treatment [39]. Avoiding AEs, as well as the associated costs, has been cited as the main advantage of non-operative treatment [12].

The stability-based classification system does not differentiate between ILMF types, as it is based solely on the instability criteria, which are not in dispute: “(1) any ankle fracture-dislocation, (2) any bimalleolar or trimalleolar ankle fracture, and (3) any lateral malleolar fracture with a significant TS (usually more than 1 to 2 mm increase in medial clear space measured relative to the superior clear space)” [4]. According to this classification, all ILMFs without significant TS should be further examined with a diagnostic stress-test to determine optimal treatment. If ILMFs are found to be stable, the patient can be managed non-operatively, regardless of fibular fracture location. Most studies comparing surgical versus non-operative treatment differentiate between infra-syndesmotic (AO44A1), trans-syndesmotic (AO44B1) and supra-syndesmotic (AO44C1/2) type ILMFs, based on the fibular fracture location in respect to the level of the anterior tibiofibular ligament and posterior inferior tibiofibular ligament. This differentiation is based on the theoretical assumption that supra-syndesmotic injuries are associated with a higher degree of instability and therefore often require surgical treatment including trans-syndesmotic fixation [2, 4].

The results of this study should be interpreted in the light of the inherent limitations of the retrospective design. Retrospective review is subject to misclassification bias. The included patients did not undergo a standardized stress-test; accordingly, the stability of the included displaced ILMFs was unknown and might not reflect the stability profile of displaced ILMFs in the target population. Preoperative computed tomography scans were available in only few of the included cases, and some patients included in this study could have had posterior malleolar fractures that were missed in standard radiographic views. We aimed to address misclassification bias and interobserver inconsistencies by including well defined variables and having multiple observers complete data capture, perform radiographic measurements and classify AEs. We believe the use of standardized AE reporting with the OrthoSAVES system is a strength of this study.

This is a non-comparative descriptive cohort study from a single center. We should be very careful in using results from single-center studies to guide clinical practice. Our study should not be seen as more than hypothesis generating and should prompt further prospective investigations into the indications for surgical treatment of displaced ILMFs. The implications of these results indicate that during the study period approximately 10% (108/1006) of all patients with surgically treated ankle fractures in our setting could potentially have been managed non-operatively, with a lower risk of AEs and a reduced need for additional surgical procedures.

## Conclusion

This study supports the notion that surgical fixation of ILMFs is not without risk of severe AEs and many patients will require later implant removal. Further prospective studies are required to ascertain whether non-operative treatment of stable displaced ILMFs can lower the risk of AEs and reduce the need for additional surgical procedures.

## Supplementary Information

**Additional file 1: Appendix**: Orthopaedic Surgical Adverse Events Severity (OrthoSAVES) System Categories [26].

## Data Availability

The datasets used and/or analyzed during the current study are available from the corresponding author on reasonable request.
