# Supplementary material for: Surgical treatment of displaced isolated lateral malleolar fractures: incidence of adverse events requiring revision: a retrospective cohort study
Source: J Orthop Surg Res. 2022 May 3;17:252. doi: 10.1186/s13018-022-03135-z (PMC9066775; doi:10.1186/s13018-022-03135-z)
Supplement: Supplementary file 1 — Additional file 1: Appendix: Orthopaedic Surgical Adverse Events Severity (OrthoSAVES) System Categories [26]. [file 13018_2022_3135_MOESM1_ESM.docx]

**Appendix: Orthopedic Surgical Adverse Events Severity System Categories**

| **Orthopedic Surgical Adverse Events Severity System Categories** [25] | |
| --- | --- |
| Intraoperative | Postoperative |
| (1) Airway/ventilation  (2) Allergic reactions  (3) Cardiac arrest/failure/arrhythmia  (4) Compartment syndrome  (5) Cutaneous injury (e.g., pressure sore)  (6) Dural tear  (7) Hypotension (clinically relevant)  (8) Implant/instrumentation related  (a) Instrumentation/fixation/implant/mal positioning requiring revision  (b) Peri-implant fracture  (9) Incorrect operative site  (10) Blood loss >5 L in 24 hrs or >2 L in 3 hrs  (11) Neural injury  (a) Spinal cord  (b) Nerve root  (c) Peripheral nerve  (12) Soft-tissue injury/failure  (a) Ligament/tendon injury requiring additional surgery  (b) Soft-tissue reconstruction/repair failure requiring revision  (13) Vascular injury  (14) Other | (15) Airway/breathing  (16) Cardiac arrest/failure/arrhythmia  (17) Cerebrovascular event  (18) Compartment syndrome  (19) Cutaneous injury (e.g., pressure sore)  (20) Delirium/altered mental state  (21) Dysphagia/dysphonia  (22) Fall  (23) Gastrointestinal bleeding  (24) Hematoma  (25) Ileus/bowel obstruction  (26) Implant/instrumentation-related  (a) Loss of reduction/alignment/correction  (b) Peri-implant fracture  (c) Joint instability/dislocation  (d) Aseptic loosening  (27) Infection  (a) Superficial wound  (b) Deep wound  (c) Urinary tract  (d) Systemic  (28) Myocardial infarction  (29) Neurological deterioration  (a) Cord (≥1 motor grade in American Spinal Injury Association [ASIA] motor scale)  (b) Nerve root/peripheral nerve ≥1 Medical Research Council (MRC) grade  (c) Cauda equina syndrome  (30) Nonunion/malunion  (31) Pain – new onset (e.g., neuropathic pain/reflex sympathetic dystrophy/pain disorder)  (32) Pneumonia  (33) Renal insufficiency  (34) Thromboembolic event  (a) Deep vein thrombosis  (b) Pulmonary embolism  (35) Soft-tissue reconstruction/repair failure  (36) Wound dehiscence  (37) Urinary retention  (38) Wound drainage (clinically significant)  (a) Cerebrospinal fluid leak/meningocele  (b) Serous (requiring treatment)  (39) Other |
